# Do Synovial Inflammation and Meniscal Degeneration Impact Clinical Outcomes of Patients Undergoing Arthroscopic Partial Meniscectomy? A Histological Study

**DOI:** 10.3390/ijms23073903

**Published:** 2022-03-31

**Authors:** Eleonora Olivotto, Elisa Belluzzi, Assunta Pozzuoli, Augusto Cigolotti, Manuela Scioni, Steven R. Goldring, Mary B. Goldring, Pietro Ruggieri, Roberta Ramonda, Brunella Grigolo, Giovanni Trisolino, Marta Favero

**Affiliations:** 1 RAMSES Laboratory, RIT Department, IRCCS Istituto Ortopedico Rizzoli, 40136 Bologna, Italy; eleonora.olivotto@ior.it (E.O.); brunella.grigolo@ior.it (B.G.); 2 Musculoskeletal Pathology and Oncology Laboratory, Orthopaedic and Traumatologic Clinic, Department of Surgery, Oncology and Gastroenterology (DISCOG), University of Padova, 35128 Padova, Italy; assunta.pozzuoli@unipd.it; 3 Orthopedics and Orthopedic Oncology, Department of Surgery, Oncology and Gastroenterology (DiSCOG), University-Hospital of Padova, 35128 Padova, Italy; augusto.cigolotti@aopd.veneto.it (A.C.); pietro.ruggieri@unipd.it (P.R.); 4 Department of Statistical Sciences, University of Padova, 35121 Padova, Italy; manuela.scioni@unipd.it; 5 Hospital for Special Surgery, Weill Cornell Medical College, New York, NY 10021, USA; sgoldring@me.com (S.R.G.); mbgoldring@me.com (M.B.G.); 6 Rheumatology Unit, Department of Medicine (DIMED), University of Padova, 35128 Padova, Italy; roberta.ramonda@unipd.it (R.R.); faveromarta@gmail.com (M.F.); 7 Pediatric Orthopedic and Traumatology, IRCCS Istituto Ortopedico Rizzoli, 40136 Bologna, Italy; giovanni.trisolino@ior.it; 8 Medicina Interna I, Ca’ Foncello Hospital, 31100 Treviso, Italy

**Keywords:** arthroscopic partial meniscectomy, synovial inflammation, meniscal degeneration, pain, clinical outcomes, osteoarthritis

## Abstract

The menisci exert a prominent role in joint stabilization and in the distribution of mechanical loading. Meniscal damage is associated with increased risk of knee OA. The aim of this study was to characterize the synovial membrane and meniscal tissues in patients undergoing arthroscopic partial meniscectomy for meniscal tear and to evaluate association with clinical outcomes. A total of 109 patients were recruited. Demographic and clinical data were collected. Visual Analogic Scale (VAS) measuring pain and Knee injury and Osteoarthritis Outcome Score (KOOS) were recorded at baseline and at 2-years follow-up. Histological and immunohistochemical characterizations were performed on synovial membranes and meniscal tissues. More than half of the patients demonstrated synovial mononuclear cell infiltration and hyperplasia. Synovial fibrosis was present in most of the patients; marked vascularity and CD68 positivity were observed. Inflammation had an impact on both pain and knee symptoms. Patients with synovial inflammation had higher values of pre-operative VAS and inflammation. Higher pre-operative pain was observed in patients with meniscal MMP-13 production. In conclusion, multivariate analysis showed that synovial inflammation was associated with pre-operative total KOOS scores, knee symptoms, and pain. Moreover, meniscal MMP-13 expression was found to be associated with pre-operative pain in multivariate analysis. Thus, targeting inflammation of the synovial membrane and meniscus might reduce clinical symptoms and dysfunction at the time of surgery.

## 1. Introduction

Knee Osteoarthritis (OA) represents the most common form of joint disease and a major cause of pain and disability in the adult population [1]. The definition of OA has changed dramatically over the past decades, and OA is no longer considered a disease involving only cartilage, but is now recognized as a disorder that might affect all joint components, including the meniscus, synovial membrane, infrapatellar fat pad, and subchondral bone [2,3,4]. The menisci play a critical protective role in joint stabilization and in the transfer and distribution of mechanical loading. Importantly, the integrity of the meniscus is key to joint health, and meniscal damage is associated with an increased risk of OA [5,6,7]. Untreated meniscal tears can cause pain, joint swelling, recurrent mechanical instability leading to significant reduction in quality of life, predominately in young and active patients [8]. Clinical studies have shown the beneficial effects of meniscal preservation compared to total meniscectomy and increased degenerative changes were identified in knees after total meniscectomy compared with those after partial meniscectomy [9]. Synovial inflammation and joint effusions often occur after meniscal injuries as demonstrated by MRI studies [10,11,12,13,14]. Scanzello et al. reported a relationship between the presence of microscopic synovitis and knee symptoms in a small cohort of patients, without any radiographic signs of OA, undergoing meniscectomy for traumatic meniscal tear [15]. There is additional evidence that synovial inflammation plays a critical role in the severity of OA symptoms and structural progression, as well as cartilage degeneration rate and osteophytosis appearance [16].

However, it is still not completely clear whether there is an association between microscopic synovial inflammation and knee pain in patients with meniscal tears. Moreover, no previous studies in the literature have investigated the histological features of both synovial and meniscal tissues in order to unravel the possible associations between pre-operative status and post-operative outcome.

Therefore, the aim of this study was to characterize both microscopic features of these tissues in patients undergoing arthroscopic partial meniscectomy (APM) for meniscal tear in relation to pre-operative status and post-operative outcomes. We hypothesized that the presence of both synovial inflammation and meniscal degeneration would be associated with enhanced clinical symptoms including increased knee pain.

## 2. Results

### 2.1. Patient Characteristics

Among the 80 patients included in the histological analysis, 56 (70%) were males and 24 (30%) were females with a median age of 47 years, interquartile range (IQR) 55.17–39.22. The median BMI of the patients was 27.45 (29.95–23.57), kg/m^2^ (IQR) (Supplementary Table S1). Macroscopic cartilage lesions were present in 57 of 78 patients (73%) in whom the cartilage scoring was complete (Figure S1).

### 2.2. Synovial Inflammation Features

The histological analysis was performed on 74 patients and 6 synovial samples were discarded due to the small size of the biopsies. The intra-reader reliability was good for mononuclear cell infiltration (0.74) and was fair for all other histological features (hyperplasia 0.56, vascularity 0.40 and fibrosis 0.42). The inter-reader reliability was moderate for mononuclear cell infiltration (0.63) and hyperplasia (0.64) and was fair for vascularity (0.55) and poor for fibrosis (0.35). These findings are in line with data available in the literature [17]. A total of 36 patients (48.6%) did not display any perivascular mononuclear cell infiltration in the synovial tissue.

**Figure 1:**
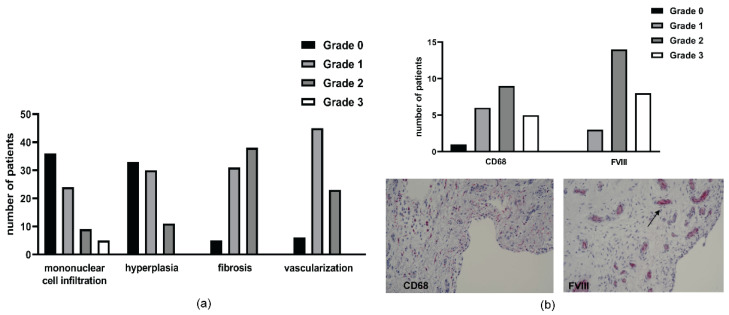
Histological and immunohistochemical analysis of synovial tissue. (**a**) synovial inflammation evaluated according to the histological synovial scoring system considering the following parameters graded from 0 to 3: mononuclear cell infiltration, hyperplasia, fibrosis and vascularization. (**b**) in the upper part: number of patients with different grades of production of CD68 and FVIII; bottom part: a representative patient exhibits high-grade CD68 production (10× magnification) and FVIII (grade 3, 20× magnification; the arrow points to immunohistochemical staining for factor VIII-related antigen, the marker for endothelial cells of vessels).

Among the samples with perivascular mononuclear cell infiltration (38 patients), most patients showed a low-grade synovitis (grade 1 = 24 patients (63.2%), grade 2 = 9 patients (23.7%)) and only 5 patients (13.2%) displayed a high grade of synovitis (grade 3). Most of the synovial specimens showed signs of synovial hyperplasia, with 30 cases (40.5%) displaying grade 1 and 11 patients (14.9%) grade 2. A total of 33 patients (44.6%) exhibited a normal lining. Synovial fibrosis was prevalent and only 5 patients (6.8%) did not show any sign of fibrosis. Vascularity was increased in more than 90% of the patients (Figure 1a).

In 20 of the 21 patients analyzed, the presence of monocytic infiltration in the synovial membrane (CD68 positive cells) was detected and all of the 25 patients assessed were positive for FVIII (Figure 1b).

### 2.3. Meniscal Features

**Figure 2:**
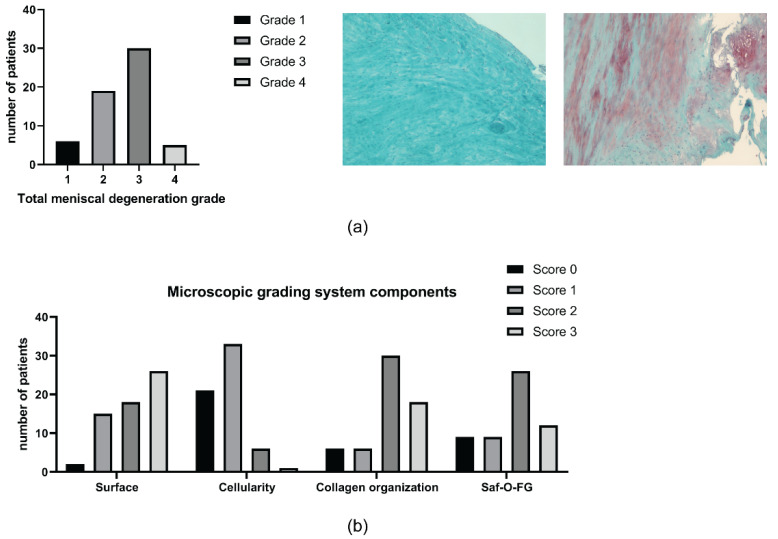
Histological meniscal degeneration grade. (**a**) On the left, total meniscal degeneration grade (from 1 to 4) and, on the right, representative images of Safranin O/Fast Green staining of grade 1 and 3 samples, respectively (magnification 10×); (**b**) Microscopic grading system components: surface, cellularity, collagen organization, and Safranin O/Fast Green stain (score from 0 to 3 for each component). Saf-O-FG = Safranin O/Fast Green stain.

A total of 6 patients (10%) of 60 analyzed did not show any signs of meniscal degeneration (grade 1). Most of the patients displayed a range from mild (grade 2) (*n* = 19, 31.7%) to moderate (grade 3) (*n* = 30, 50%) meniscal degeneration and five patients (8.3%) presented a severe grade of meniscal degeneration (grade 4). Analysis of the meniscal degeneration grading scores is shown in Figure 2a. The histological meniscal degeneration grade was assessed by a microscopic grading system, which evaluates changes in surface, cellularity, collagen organization, and Safranin O/Fast green staining. For each single component related to surface and matrix degradation, only a few patients showed grade 0; on the contrary, all patients showed signs of degradation. Interestingly, most of the patients showed normal to increased diffuse cellularity. Analysis of the histological grading scores is shown in Figure 2b.

**Figure 3:**
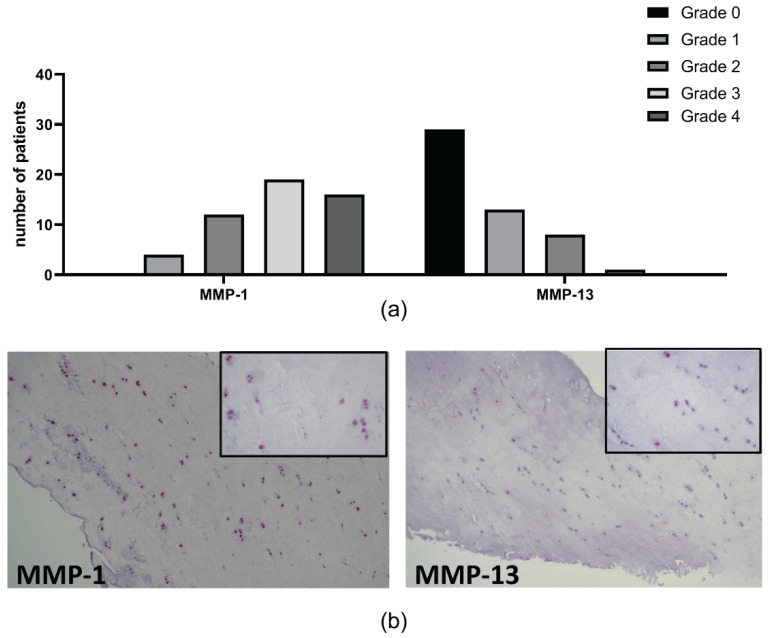
Immunohistochemistry of metalloproteinase in meniscal samples. (**a**) number of patients with different expression of MMP-1 and -13; (**b**) one representative patient for MMP-1 (grade 3) and MMP-13 (grade 1), (magnification 10×, inset 40×, respectively).

**Figure 4:**
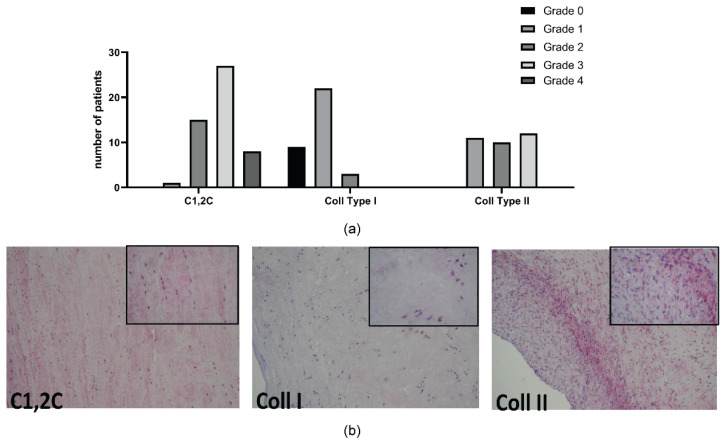
Immunohistochemistry of collagens in meniscal samples. (**a**) number of patients with different expressions of C1,2C, Coll Type I and II; (**b**) one representative patient for each marker (grade 3, 2, 3, respectively) (magnification 10×, inset 40×, respectively).

Immunohistochemical analysis was performed in 51 patients to investigate the protein expression of metalloproteinase (MMP)-1, MMP-13, C1,2C fragment, Collagen type I, and Collagen type II (Figure 3 and Figure 4).

All samples expressed MMP-1, mostly with strong signal in almost the entire section analyzed. On the contrary, most of the samples analyzed (57%) did not show any signal for MMP-13, while the remaining showed a grade 2 or 3 score in almost the entire section analyzed (Figure 3). Regarding C1,2C, all samples expressed the collagen neoepitope mostly with strong signal (Figure 4).

As C1,2C is the neoepitope of the three-quarter length piece generated by the cleavage of types I and II collagens by collagenases, the presence of the collagen type I in 34 and collagen type II in 33 samples was analyzed. Collagen type II was present in all samples, while collagen type I was absent in 27% of the samples (Figure 4).

### 2.4. Clinical Outcomes

**Figure 5:**
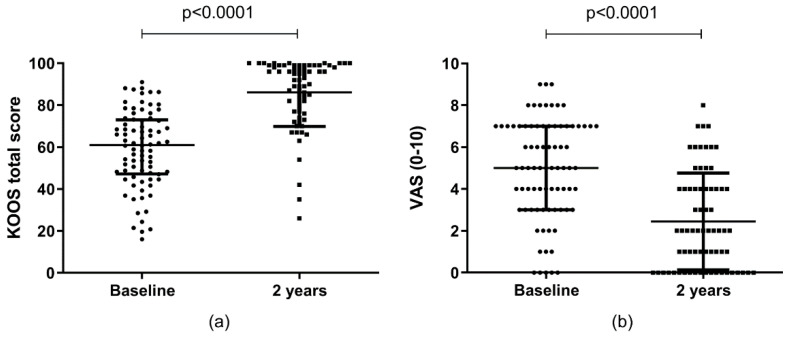
Comparison between KOOS total score (**a**) and VAS (**b**) at the baseline and 2 years after surgery. Data are reported as median and interquartile range.

All enrolled patients completed visual analogue score (VAS) pain scale and Knee Injury and Osteoarthritis Outcome Score (KOOS) questionnaires at the time of the surgery, while 11 patients were absent at the 2 year follow-up. The KOOS total score improved from a median of 61.01 at the baseline to a median of 91.67, 2 years post-surgery (*p* < 0.0001) (Figure 5a). VAS pain improved from a median of five at the baseline to two, 2 years post-surgery (*p* < 0.0001) (Figure 5b).

Patients with the presence or absence of synovial mononuclear cell infiltration were compared in order to evaluate whether this variable impacted on the clinical outcomes. No difference was observed related to age, body mass index (BMI) or time to surgery between the two groups. An overall deterioration of the pre-operative knee function with the presence of synovial mononuclear cell infiltration was observed (Table 1). Patients with synovial mononuclear cell infiltration showed a statistically significant worsening of total KOOS scores compared to patients without infiltration (*p* = 0.015). Even KOOS symptoms and pain subscales were lower in patients with mononuclear cell infiltration (*p* = 0.006 and 0.002, respectively). Conversely, the KOOS Activities of Daily Living (ADL) subscale was higher in patients without synovial mononuclear cell infiltration (*p* = 0.045).

The pre-operative VAS score was worse in patients with synovial mononuclear cell infiltration compared with patients without infiltration (*p* = 0.005). At 2 years follow-up, no difference was observed between the two groups (Table 1). Differences between the two groups were observed regarding the delta (∆ = post-operative–pre-operative) KOOS pain subscale (*p* = 0.032) and ∆VAS (*p* = 0.019) (Table 1).

No association was found between the other histological features of the synovial inflammation score (hyperplasia, fibrosis, and vascularity) and pre- or post-operative KOOS and VAS (data not shown).

**Figure 6:**
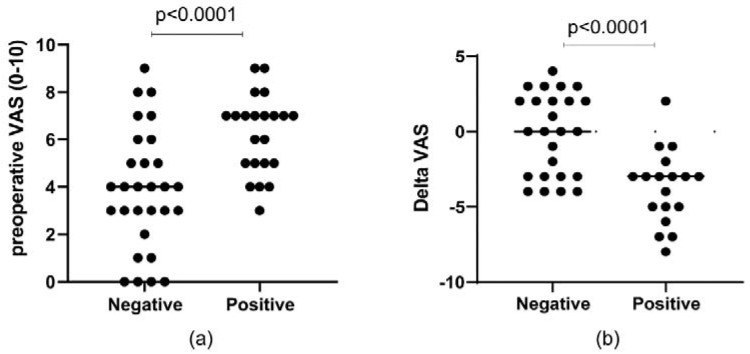
Plots showing the preoperative (**a**) and delta VAS (**b**) in patients with negative or positive MMP-13.

Regarding structural changes in meniscal tissues, no difference was observed comparing pre- and post-operative KOOS and VAS with respect to the different histological features (surface, cellularity, collagen organization, degeneration score) or immunohistochemistry MMP-1, MMP-13, Collagen type I, Collagen type II, and Collagen C1, 2C) (data not shown). However, higher pre-operative VAS and a lower ∆VAS were observed in patients with positive MMP-13 expression (*p* < 0.0001) (Figure 6).

### 2.5. Correlations

**Figure 7:**
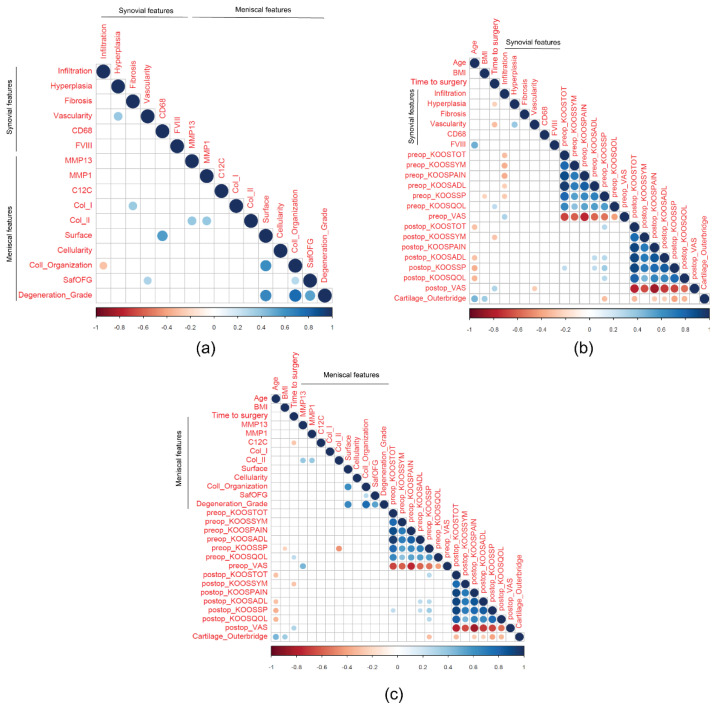
Correlations between tissues and clinical data. (**a**) Correlations between synovial and meniscal tissues; (**b**) Correlations between synovial features and clinical data; (**c**) Correlations between meniscal and clinical data. Negative correlations are displayed in red, while positive correlations are in blue. Color intensity and size of the circle are proportional to the correlation coefficients.

Correlations between tissue analysis and clinical data are reported in Figure 7. A moderate positive correlation was observed between meniscal surface integrity and CD68 positive cells in synovial tissue (r = 0.555, *p* = 0.017). Integrity of meniscal surface was highly positively correlated with collagen organization (r = 0.626, *p* < 0.0001).

Meniscal surface, collagen organization and S-O-FG staining positively correlated with meniscal degeneration score (0–4) (r = 0.654, *p* < 0.0001; r = 0.777, *p* < 0.0001, and r = 0.534, *p* < 0.0001, respectively) (Figure 7a).

Age positively correlated with synovial FVIII positive cells (r = 0.487, *p* = 0.013) and with the cartilage Outerbridge score (r = 0.470, *p* < 0.0001). Moreover, age negatively correlated with post-operative KOOS total score (r = −0.298, *p* = 0.013), ADL (r = −0.292, *p* = 0.015), sports and recreational activities (SSP) (r = −0.351, *p* = 0.0039), and QOL (r = −0.332, *p* = 0.005) subscales. BMI was observed to be negatively correlated with pre-operative KOOS total score (r = −0.221, *p* = 0.049) and positively with the cartilage Outerbridge score (r = 0.358, *p* = 0.001). Time to surgery negatively correlated with synovial hyperplasia (r = −0.234, *p* = 0.045), synovial vascularity (r = −0.305, *p* = 0.008), and C1,2C epitopes in the meniscus (r = −0.277, *p* = 0.049). The cartilage Outerbridge score was negatively correlated with postoperative KOOS total score (r = −0.330, *p* = 0.006), pain subscale (r = −0.265, *p* = 0.030), ADL subscale (r = −0.242, *p* = 0.049), SSP subscale (r = −0.405, *p* = 0.001), and QOL subscale (r = −0.336, *p* = 0.006).

Synovial mononuclear cell infiltration negatively correlated with pre-operative total KOOS score (r = −0.305, *p* = 0.008), symptoms (r = −0.360, *p* = 0.002), ADL (r = −0.248, *p* = 0.033), and sport (r = −0.265, *p* = 0.022) subscales, and pre-operative VAS (r = 0.315, *p* = 0.006) (Figure 7b). Meniscal MMP-13 positively correlated with pre-operative VAS (r = 0.461, *p* = 0.001) (Figure 7c).

A moderately positive correlation was observed between meniscal surface integrity and CD68-positive cells in synovial tissue (r = 0.555, *p* = 0.017). Integrity of the meniscal surface was highly correlated with collagen organization (r = 0.626, *p* < 0.0001). Meniscal surface, collagen organization, and S-O-FG staining correlated with meniscal degeneration score (0–4) (r = 0.654, *p* < 0.0001; r = 0.777, *p* < 0.0001, and r = 0.534, *p* < 0.0001, respectively).

### 2.6. Multivariate Analysis

Multiple linear regression analysis was performed to determine whether the relationships between clinical outcomes and synovial mononuclear cell infiltrate were independent from known OA risk factors. Age, Outerbridge “total score”, BMI, and time between injury and surgery were included as independent covariates. The presence of synovial mononuclear cell infiltration was found to be independently associated with pre-operative KOOS total score (*p* = 0.0286), pre-operative KOOS symptoms subscale (*p* = 0.0170), pre-operative KOOS pain subscale (*p* = 0.0033), and pre-operative VAS (*p* = 0.0055) (Table 2 and Table S2), but not with post-operative KOOS and VAS at 2 years follow-up (data not shown).

The same models were applied also to identify independent associations among clinical outcomes (∆KOOS total score and subscales ∆VAS) and synovial infiltration and risk factors. The presence of synovial mononuclear cell infiltration was observed to be independently associated with ∆KOOS total score (*p* = 0.0424), ∆KOOS pain subscale (*p* = 0.0145), and ∆VAS (*p* = 0.0189) (Table 3 and Table S3). The presence of vascularization was independently associated with ∆VAS (*p* = 0.0437). No other association was found between the other synovial histological features and the clinical outcomes (data not shown).

Regarding the meniscal features, only MMP-13 was observed to be independently associated with pre-operative VAS (*p* = 0.002) and Δ VAS (*p* < 0.001) (Table 4).

## 3. Discussion

The meniscus plays an important role as a joint stabilizer and it is well established that patients with meniscal tears have a higher risk of developing OA [18]. There is a strong association between degenerated menisci and OA [19] and recent evidence suggests that the meniscus also plays a biological role in OA [20,21,22] through the increased production of pro-inflammatory mediators and matrix-degrading enzymes [20,21,22,23].

In our study, only 27% of the patients had normal cartilage recorded during arthroscopic surgery. Based on the literature that highlight the importance of the meniscus in OA onset and progression [24], we analyzed not only the impact of synovial inflammation, which was observed both in early and late stages of OA [20,25], but also the potential influence of meniscal structural changes both on pre-operative status and post-operative clinical outcomes.

The healthy synovial membrane is composed of two layers: the intima, which lies next to the joint cavity and consists of a layer of 1–4 cells, only 20–40 μm thick and the subintima that can be up to 5 mm [16,26]. More than half of the patients demonstrated synovial mononuclear cell infiltration and hyperplasia. Synovial fibrosis was present in most of the patients and marked vascularity was observed in more than 80% of the patients. In OA patients, the synovial membrane is characterized by synovial lining hyperplasia, sublining fibrosis, stromal vascularization and macrophage and T-cell lymphocytes infiltration [27]. The same histological features can be also found in the early phase of inflammatory arthritis, even if they are more prominent, whereas organized lymphoid follicles and pannus tissue are features generally associated with inflammatory arthritis [28].

All patients showed cells positive to FVIII. In addition, all patients except one exhibited CD68 positive cells, a macrophage marker, in the synovial infiltrates, which correlated with an overall deterioration of knee function and pain (measured through VAS scale) before surgery. Interestingly, CD68 positive cells were also found in the synovial membrane of patients affected by femoro-acetabular impingement, which predisposes patients to hip OA [29,30]. As CD68 does not distinguish pro-inflammatory macrophages (M1) from anti-inflammatory macrophages (M2), other studies need to be performed to further investigate the macrophage phenotype.

Regarding the intra- and inter-reliability, our data are substantially in agreement with those obtained by Scanzello et al. [17]. Indeed, moderate to good intra- and inter-reliability was found for mononuclear cell infiltration and hyperplasia, while weak intra- and inter-reliability was found for fibrosis. Weak intra- and moderate inter-reliability was observed for vascularity.

Lower values of pre-operative KOOS total score were found in patients with synovial inflammation in accordance with the results obtained by Scanzello et al. [15], which used the Lysholm score, a knee-specific metric of symptoms and functional disability. Lysholm is a weighted score, with pain and instability-related symptoms having the most weight. In contrast to Scanzello et al. [17], in our study we used the KOOS score to possess the possibility to evaluate the impact of the synovial inflammation and meniscal changes on the different subscales. We observed that patients with synovial inflammation had lower pre-operative KOOS symptoms and pain subscales suggesting that inflammation had an impact on both pain and knee symptoms based on univariate analysis. Moreover, negative correlations were observed between synovial inflammation and pre-operative KOOS scores. Patients with synovial infiltrate had higher values of pre-operative VAS and synovial infiltrate correlated positively with the pre-operative VAS scores. On the contrary, no association was observed in the study of Scanzello et al. [17].

Multivariate analysis showed that synovial inflammation was associated with pre-operative KOOS total score, pre-operative KOOS symptoms and pain subscales, and pre-operative VAS independently from the cartilage Outerbridge score, duration of symptoms, BMI or age. An improvement of post-operative KOOS and VAS scores was observed in patients with and without synovial inflammation, but no difference was observed when comparing the clinical outcomes between the two groups. These results agree with those obtained by Scanzello et al. [17]. A difference in the ∆KOOS pain subscale and ∆VAS was observed in comparing patients with or without synovial inflammation, while we identified a borderline difference for ∆KOOS total score (*p* = 0.057) in univariate analysis. Multivariate analysis confirmed all the associations (including ∆ total KOOS). On the contrary, Scanzello et al. found an impact of synovial inflammation only on ∆16 week Lysholm scores and not ∆2 year Lysholm scores [17]. The impact of synovial inflammation on knee symptoms in patients with meniscal tears was not surprising, as it has already been reported by previous studies [15,17]. The new and important finding in our cohort of patients is the association of synovial inflammation with pre-operative pain, as already reported in late OA [3].

As predicted, only very few patients showed meniscal integrity (6 out of 60 analyzed) in line with previously reported findings [31,32]. Regarding the components of the microscopic grading system, most of the patients showed slight to severe fibrillation of the surface, diffuse cellularity, collagen fibers unorganized, severe fraying and tears, and moderate safranin O staining.

Immunohistochemical analysis of MMP-1 expression revealed that it was highly expressed in all samples, while a strongly positive signal for MMP-13 was observed in approximately 50% of the samples. The presence of MMP-1 expression is not surprising since it has been shown that pro-inflammatory stimuli induce high expression levels of this metalloproteinase in meniscal cells [21].

Importantly, in univariate analysis we discovered higher pre-operative pain scores in patients with positive MMP-13 expression. This metalloproteinase is one of the key effectors in the cartilage degradation network in OA [33]. Brophy et al. reported a higher expression of MMP-13 in younger patients with meniscal tears compared to patients over 40 years with meniscal tears [34], suggesting that age bears an impact on MMP-13 expression. In our study, multivariate analysis confirmed the association of MMP-13 expression with pain using age as a covariate. As reported in the literature, in animal models of OA, the detection of increased expression levels of MMP-13 suggests that this metalloproteinase may be an important factor involved in the early-onset of OA joint pathology [33]. We did not observe an impact of MMP-13 expression on post-operative clinical outcomes after 2 years follow-up, but it is possible that a longer follow-up is needed to detect this effect.

As confirmed by the presence of MMP-related activity, the carboxy terminus neoepitope C1,2C generated by the cleavage of types I and II collagens was found in all meniscal samples indicating a high rate of cleaved collagens [35,36]. As shown by immunohistochemistry, the meniscal tissues were mainly composed of collagen type II, while collagen type I was present in 74% of the samples. This is in line with the normal composition and microstructure of the meniscus, in which the inner avascular zone, identified as the white-white region, is mainly composed of collagen type II [8,37].

In the future, it would be interesting to investigate the potential involvement in early OA of the following cytokines such as IL-15 [38], IL-17 [39,40,41], IL-18 [42], IL-22 [39], IL-23 [40], and IL-33 [42,43].

Interestingly, a recent study published by Grammens et al. observed a link between a small medial condyle and medial compartment degeneration in 16 patients [44]. Thus, it would be important to explore whether the knee morphotype impacts on clinical outcome of patients undergoing APM.

The main limitations of our study were: the medium-term follow-up of two years; the lack of data on OA progression of the patients hindering the possibility to investigate possible associations between synovial and meniscal pathology and OA development. Nevertheless, the clinical outcomes of the patients were evaluated using KOOS score, a wide range score that enables assessment of different aspects of knee functionality and the impact on the quality of life. In addition, the synovial membrane was collected only in the suprapatellar compartment, representing the most frequent site affected by synovial inflammation in patients with meniscal tear; nevertheless, this region may not be representative of the entire joint. Moreover, the meniscus samples were collected mostly from the inner region which is well known to bear different features from the outer zone. We did not find evidence of a relationship between synovial inflammation and the clinical findings at the 2-year follow-up, which may be due to the small sample size and to the short duration of the follow-up. Finally, we did not possess information concerning pain medications used by the patients before surgery, which could have influenced pain assessment.

Nevertheless, the strengths of our study are in the large number of patients enrolled compared to other studies, the comprehensive characterization of the joint pathology and clinical features in our cohort of patients. In addition, we included the characterization of not only the synovial membrane pathology but also of the meniscal pathology, using the KOOS scoring system, which was shown to be a reliable and valid questionnaire that enabled the assessment of each clinical variable using individual scores [45].

## 4. Materials and Methods

### 4.1. Patient Recruitment and Clinical Data Collection

A total of 109 patients (74 men [67.89%] and 35 women [32.11%], mean age ± [SD] 47.45 ± [11.03] years) undergoing APM for symptomatic degenerative or traumatic meniscal tears were recruited within the framework of a multicenter prospective cohort study funded by the Italian Ministry of Health. This observational study was approved by the IRCCS Istituto Ortopedico Rizzoli and Padova Hospital Ethical Committees. Patients were enrolled after providing written informed consent and meeting eligibility criteria, as described in our previous study [46]. Moreover, patients undergoing meniscal repair were excluded from the present study, since meniscal tissue could not be removed, and to reduce potential confounders affecting the final outcomes. At the baseline and at the 2-year follow-up, the following pre-operative clinical data were collected: age, sex, BMI, date of injury and time to surgery, history of trauma, pre-operative VAS measuring pain [47], and KOOS. The KOOS questionnaire, which was developed with the purpose of evaluating short- and long-term symptoms and function in subjects with knee injury and OA, is self-administered and assesses five outcomes: pain, symptoms, activities of daily living (ADL), sport and recreation function (Sport/Rec), and knee-related quality of life (QOL). Standardized answer options are given (5 Likert boxes) and for each question a score from 0 to 4 is assigned. A normalized score is calculated for each subscale (100 indicating no problems and 0 indicating extreme problems). The KOOS outcome measure was translated and validated in Italian language [48].

Cartilage damage was assessed during arthroscopic surgery in the following six different knee compartments: medial and lateral femoral condyles, medial and lateral tibial plateau, trochlea and patella using Outerbridge grading system [49]. The most severe Outerbridge score in each of the compartments was considered.

### 4.2. Tissue Sample Collection

From a total cohort of 109 patients, meniscal tissue was retrieved during arthroscopic surgery from 80 patients undergoing partial meniscectomy for symptomatic meniscal tears. Moreover, to avoid excessive bleeding, only a small specimen of synovial tissue was collected from the suprapatellar compartment. The suprapatellar pouch is the most visually accessible and the most common site to detect synovial inflammation during arthroscopic meniscectomy [15].

### 4.3. Histological Analyses

Joint tissues were fixed in 4% formalin, dehydrated in 70% ethanol, and embedded in paraffin. Sections of 5 μm were cut using a rotator microtome (Leika Biosytems RM2255, Milano, Italy), deparaffinized in xylene and rehydrated in ethanol for histological and immunohistochemical analyses. One experienced biologist performed all the observations and ratings.

To evaluate the synovial inflammation, the sections were stained with hematoxylin-eosin (H&E) (Bioptica, Milano, Italy) and observed at ×20 magnification. Only sections containing a clearly recognizable synovial lining layer with underlying vascularized subintima were evaluated. The synovial inflammation was evaluated according to the histological synovial scoring system used by Scanzello and colleagues [16,17,50]. To evaluate inter- and intra-reader reliability, 42 synovial specimens were scored by two independent readers (E.O., M.F.) and 20 were re-scored by one blinded reader (E.O.). When the synovial scoring was different between readers, slides were re-evaluated by both readers in order to achieve an agreement.

The degree of meniscal degeneration was assessed by a modified Pauli’s microscopic grading system composed of 4 categories (I–surface, II–cellularity, III–collagen organization/alignment and fiber organization and IV–matrix staining) as already reported in Battistelli et al. [32] and Trisolino et al. [29] and specified in Supplementary Table S4. To evaluate the proteoglycan/collagen content the sections were stained with H&E and 0.25% Safranin-O/0.3% Fast Green (S-O-FG) (Sigma Aldrich, St. Louis, MO, USA) and observed at ×10 magnification. After the evaluation of each category, the total grade of meniscal degeneration was calculated (adding the scores obtained in each category) as follows: 1 = normal tissue (scores 0–3); 2 = mild degeneration (scores 4–6); 3 = moderate degeneration (7–9) and 4 = severe degeneration (10–12).

### 4.4. Immunohistochemical Analyses

The monocyte infiltration in the synovial membrane samples was characterized with a CD68 monoclonal primary antibody (M0814, DAKO, Santa Clara, CA, USA), while the vascularity was evaluated using the polyclonal Anti-Factor VIII-Related Antigen antibody (Anti-von Willebrand Factor, A0082, DAKO, Santa Clara, CA, USA). Positive staining for each antibody for each section was semi-quantitatively evaluated according to the following criteria: (a) CD68: grade (G): 0 = no signal, G1 = from 1 to 5 positive cells, G2 = from 6 to 20 positive cells, G3 > 20 positive cells; (b) Factor VIII: G0 = no signal, G1 = from 1 to 5 positive vessels, G2 = from 6 to 20 positive vessels, G3 > 20 positive vessels.

In the meniscal samples, the presence of the following proteins was evaluated: metalloproteinase (MMP)-1 (MAB3307, Millipore, Burlington, MA, USA), MMP-13 (MAB511, R&D, Minneapolis, MN, USA), C1,2C also called Col 2 ¾C Short (50-1035, IBEX, Montreal, Quebec, Canada) which is the carboxy terminus neoepitope of the three-quarter length piece generated by the cleavage of types I and II collagens by collagenases, and Collagen Type I and II (MAB3391 and MAB8887, Millipore, Burlington, MA, USA) using specific monoclonal and polyclonal antibodies. The presence of the proteins was detected using a streptavidin–enzyme conjugated system (4 + Universal AP Detection kit) and the substrate Vulcan Fast Red Chromogen kit 2 (AP506US and FR805S, Biocare Medical, Pacheco, CA, USA). Isotype-matched immunoglobulin (IgG1 and IgG2a MAB002-3, R&D; rabbit serum (normal) (X0902, DAKO, Santa Clara, CA, USA) was used as negative controls (Supplementary Figure S2). Results are expressed as the mean of positive cells per section, with 2 to 3 sequential sections analyzed for each patient. Positivity for each protein for each section was semi-quantitatively evaluated according to the following criteria. (a) MMP-1 and -13: G0 = no signal, G1 = few cells with weak positivity, G2 = few cells positive, G3 = 90% positive cell with strong signal; G4 = 100% positive cells; (b) C1,2C: G0 = no signal, from G1 to 4 increasing intensity and diffusion of positive signal in the extracellular matrix; (c) Coll Type I and II: G0 = no signal, from G1 to 3 increasing intensity and diffusion of positive signal in the extracellular matrix. All images were captured using a Nikon Eclipse 90i microscope equipped with Nikon Imaging Software elements.

### 4.5. Statistical Analysis

Results are reported as median and interquartile range (IQR) for continuous variables, while the number and percentage of patients are reported for categorical variables. Continuous variables were tested for normality using the Shapiro–Wilk test. Intra and inter-rater reliability were tested using linear weighted Cohen’s kappa for ordinal variables. Differences between groups were evaluated with Mann–Whitney U tests or Student’s t tests depending on the data distribution. Correlations were assessed using Spearman’s correlation coefficients. Univariable and multivariable analyses with general linear models were applied to adjust for lack of independence of the data. Shapiro–Wilk test was used to determine whether residuals were distributed normally. In cases of non-normal residuals appropriate transformations of the response variable were modeled. All analyses were performed with IBM SPSS version for Windows, version 28.0 (IBM Corp., Armonk, NY, USA) or R [51]. A *p* value < 0.05 was considered as statistically significant.

## 5. Conclusions

In conclusion, we show that synovial inflammation is associated with pre-operative total KOOS scores, knee symptoms, and pain in adjusted analyses. Meniscal MMP-13 expression is associated with pre-operative pain, independent of age, cartilage degeneration, BMI, and time to surgery. Thus, targeting inflammation of the synovial membrane and meniscus might reduce clinical symptoms and dysfunction at the time of surgery. Further studies in a larger cohort of patients are needed to confirm these data in long-term follow-up.

## Figures and Tables

**Table 1:** Demographic and clinical outcomes of patients with/without synovial mononuclear cell infiltration.

	Absence of Synovial Infiltrate (36 Patients)	Presence of Synovial Infiltrate (38 Patients)	*p*-Value
Age	48.14 (56.66–35.25)	46.99 (54.08–40.91)	0.645
BMI	27.70 (31.80–23.53)	27.35 (29.92–23.72)	0.845
Time to surgery	0.77 (1.69–0.39)	0.56 (1.49–0.32)	0.455
Pre-operative			
KOOS	66.66 (79.91–53.87)	54.76 (68.45–44.64)	0.015
KOOS symptoms	76.78 (85.71–60.71)	60.71 (85.71–60.71)	0.006
KOOS pain	72.22 (82.64–58.33)	55.55 (69.44–44.44)	0.002
KOOS ADL	77.94 (93.75–55.15)	64.70 (79.78–50.00)	0.045
KOOS sport	37.50 (55.00–25.00)	22.5 (45.00–10.00)	0.061
KOOS QoL	43.75 (56.25–31.25)	40.62 (56.25–25.00)	0.557
VAS	4 (6–2)	6 (7–4)	0.005
Post-operative			
KOOS	94.05 (98.81–76.34)	91.67 (99.40–80.95)	0.747
KOOS symptoms	96.43 (100.00–82.14)	96.43 (100.00–82.14)	0.973
KOOS pain	93.06 (100.00–80.56)	97.22 (100.00–83.33)	0.840
KOOS ADL	99.27 (100.00–75.74)	97.06 (100.00–83.82)	0.868
KOOS sport	90.00 (100.00–57.50)	90.00 (100.00–65.00)	0.813
KOOS QoL	81.25 (100.00–68.75)	87.50 (100.00–75.00)	0.433
VAS	2 (5–0)	2 (4–0)	0.370
Δ KOOS	19.34 (32.89–5.51)	28.57 (42.86–23.81)	0.057
Δ KOOS symptoms	16.07 (25.89–5.35)	25.00 (35.72–14.28)	0.081
Δ KOOS pain	18.05 (31.94–2.08)	36.11 (41.67–19.44)	0.032
Δ KOOS ADL	10.29 (32.72–2.57)	22.06 (41.18–8.82)	0.124
Δ KOOS sport	42.50 (56.25–3.75)	45.00 (70.00–35.00)	0.065
Δ KOOS QoL	31.25 (56.25–18.75)	37.50 (50.00–25.00)	0.492
Δ VAS	−1.5 (−4–2)	−3 (−5–−2)	0.019

For all variables median and interquartile ranges were reported. A total of 11 patients were lost during follow-up (34 patients with absence of synovial Infiltrate and 31 with presence of synovial infiltrate). Δ = post-operative–pre-operative. KOOS = Knee Injury and Osteoarthritis Outcome Score; VAS = visual analogue score, BMI = body mass index.

**Table 2:** Multivariate linear regression models between clinical outcomes and synovial infiltrate and the other OA risk factors.

	Estimate	Std. Error	T Value	*p*-Value
Preop KOOS				
KOOS TOTAL				
(Intercept)	80.77	16.55	4.82	<0.001
Synovial infiltrate	−9.38	4.19	−2.24	0.029
Cartilage Outerbridge	0.48	0.56	−0.85	0.399
Duration of symptoms	−0.0007	0.96	−0.001	0.999
Age	0.02	0.21	0.09	0.924
BMI	−0.55	0.51	−1.06	0.291
KOOS symptoms				
(Intercept)	59.71	17.48	3.41	0.001
Synovial infiltrate	−10.84	4.43	−2.45	0.017
Cartilage Outerbridge	−0.53	0.59	−0.89	0.377
Duration of symptoms	0.26	1.02	0.26	0.795
Age	0.15	0.22	0.67	0.502
BMI	0.24	0.54	0.45	0.656
KOOS pain				
(Intercept)	87.33	16.59	5.26	<0.001
Synovial infiltrate	−12.82	4.20	−3.05	0.003
Cartilage Outerbridge	−0.01	0.56	−0.02	0.983
Duration of symptoms	−0.31	0.96	−0.32	0.746
Age	−0.06	0.21	−0.27	0.790
BMI	−0.56	0.52	−1.08	0.284
Pre-op VAS				
(Intercept)	3.06	2.22	1.38	0.172
Synovial infiltrate	1.61	0.56	2.87	0.005
Cartilage Outerbridge	0.02	0.07	0.21	0.833
Duration of symptoms	0.06	0.13	0.50	0.620
Age	0.01	0.03	0.52	0.607
BMI	0.005	0.07	0.07	0.945

KOOS = Knee Injury and Osteoarthritis Outcome Score; VAS = visual analogue score, BMI = body mass index, pre-op = pre-operative.

**Table 3:** Multivariate linear regression models between clinical outcomes (Δ KOOS and Δ VAS) and synovial infiltrate and other OA risk factors as dependent variables.

	Estimate	Std. Error	T Value	*p*-Value
Δ KOOS total score				
(Intercept)	13.13	20.82	0.63	0.531
Synovial infiltrate	10.97	5.28	2.08	0.042
Cartilage Outerbridge	−0.55	0.71	−0.77	0.443
Duration of symptoms	−1.072	1.17	−0.91	0.365
Age	−0.24	0.26	−0.94	0.352
BMI	0.77	0.64	1.21	0.232
Δ KOOS pain				
(Intercept)	−7.05	21.27	−0.33	0.742
Synovial infiltrate	13.61	5.40	2.52	0.014
Cartilage Outerbridge	−1.17	0.72	−1.62	0.111
Duration of symptoms	−0.89	1.20	−0.75	0.459
Age	−0.06	0.26	−0.24	0.811
BMI	1.22	0.65	1.87	0.067
Δ KOOS Sport				
(Intercept)	39.07	27.88	1.40	0.167
Synovial infiltrate	15.15	7.08	2.14	0.037
Cartilage Outerbridge	−0.66	0.95	−0.69	0.491
Duration of symptoms	−0.09	1.57	−0.06	0.954
Age	−0.92	0.34	−2.67	0.01
BMI	1.52	0.86	1.78	0.081
Δ VAS				
(Intercept)	0.17	3.11	0.05	0.957
Synovial infiltrate	−1.91	0.79	−2.42	0.019
Cartilage Outerbridge	0.09	0.11	0.86	0.390
Duration of symptoms	0.14	0.17	0.80	0.424
Age	−0.002	0.04	−0.06	0.955
BMI	−0.07	0.09	−0.72	0.473

KOOS = Knee Injury and Osteoarthritis Outcome Score; VAS = visual analogue score, BMI = body mass index, ∆ = post-operative–pre-operative.

**Table 4:** Multivariate analysis between clinical outcomes (VAS and Δ VAS) and the influence of MMP-13 and other variables as dependent variables.

VAS	Estimate	Std. Error	T Value	*p*-Value
Preop VAS				
(Intercept)	3.02	2.64	1.15	0.258
MMP-13	1.32	0.39	3.36	0.002
Cartilage Outerbridge	−0.01	0.08	−0.12	0.908
Duration of symptoms	−0.06	0.19	−0.32	0.749
Age	0.05	0.03	1.39	0.171
BMI	−0.04	0.08	−0.47	0.641
Δ VAS				
(Intercept)	2.60	3.50	0.74	0.463
MMP-13	−1.90	0.52	−3.62	0.001
Cartilage Outerbridge	0.12	0.11	1.03	0.291
Duration of symptoms	0.42	0.24	1.70	0.097
Age	−0.04	0.04	−1.03	0.309
BMI	−0.08	0.11	−0.73	0.471

VAS = visual analogue score, BMI = body mass index, pre-op = pre-operative, ∆ = post-operative–pre-operative.

## Data Availability

The data presented in this study are available on request from the corresponding author.

## Supplementary Materials

The following supporting information can be downloaded at: https://www.mdpi.com/article/10.3390/ijms23073903/s1.
