# Supplementary material for: Do Synovial Inflammation and Meniscal Degeneration Impact Clinical Outcomes of Patients Undergoing Arthroscopic Partial Meniscectomy? A Histological Study
Source: Int J Mol Sci. 2022 Mar 31;23(7):3903. doi: 10.3390/ijms23073903 (PMC8999499; doi:10.3390/ijms23073903)
Supplement: Supplementary file 1 [file ijms-23-03903-s001.zip › ijms-1645813-supplementary.pdf]

Supplementary Materials

Table S1. Baseline demographic and clinical characteristics of the patient cohort (80 patients)

Parameter	Value
Number Men (%)/Number women (%)	56 (70)/24(30)
Median age (IQR) years	46.99 [55.17-39.22]
Median BMI (IQR) kg/m^2	27.45 [29.95-23.57]
Right/Left Knee, number (%)	44 (55)/36 (45)
Median symptom duration (IQR) years	0.73 [1.53-0.32]

IQR = Interquartile range

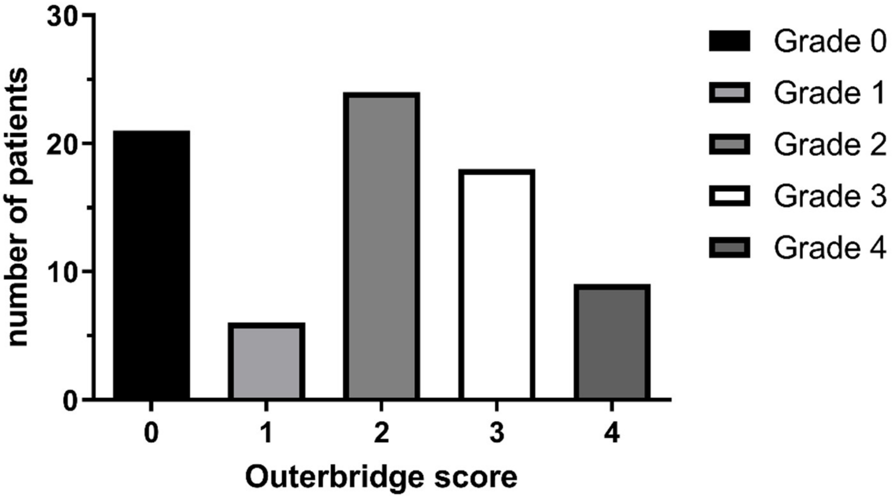

Figure S1. Cartilage scores (78 patients). The most severe cartilage damage score evaluated by Outerbridge scoring, in one of the six compartments (medial and lateral femoral condyles, medial and lateral tibial plateau, trochlea and patella) was selected. 21 patients (27%) did not show cartilage damage. 6 patients (8%) exhibited grade 1 cartilage damage, 24 patients (31%) demonstrated grade 2, 18 patients (23%) demonstrated grade 3 and 9 patients (11%) grade 4.

Table S2. Multivariate analysis between pre-operative status and synovial infiltrate and other OA risk factors

**Preop KOOS**	**Estimate**	**Std. Error**	**T Value**	**p-value**
KOOS TOTAL				
(Intercept)	80.77	16.55	4.82	<0.001
Synovial infiltrate	-9.38	4.19	-2.24	0.029
Cartilage Outerbridge	0.48	0.56	-0.85	0.399
Duration of symptoms	-0.0007	0.96	-0.001	0.999
Age	0.02	0.21	0.09	0.924
BMI	-0.55	0.51	-1.06	0.291
KOOS symptoms				
(Intercept)	59.71	17.48	3.41	0.001
Synovial infiltrate	-10.84	4.43	-2.45	0.017
Cartilage Outerbridge	-0.53	0.59	-0.89	0.377
Duration of symptoms	0.26	1.02	0.26	0.795
Age	0.15	0.22	0.67	0.502
BMI	0.24	0.54	0.45	0.656
KOOS pain				
(Intercept)	87.33	16.59	5.26	<0.001
Synovial infiltrate	-12.82	4.20	-3.05	0.003
Cartilage Outerbridge	-0.01	0.56	-0.02	0.983
Duration of symptoms	-0.31	0.96	-0.32	0.746
Age	-0.06	0.21	-0.27	0.790
BMI	-0.56	0.52	-1.08	0.284
KOOS Function, daily living				
(Intercept)	94.02	21.21	4.43	<0.0001
Synovial infiltrate	-8.70	5.37	-1.62	0.110
Cartilage Outerbridge	-0.40	0.72	-0.56	0.580
Duration of symptoms	-0.041	1.23	-0.03	0.973
Age	-0.130	0.27	-0.49	0.629
BMI	-0.51	0.66	-0.77	0.441
KOOS Sport				
(Intercept)	77.33	21.53	3.59	0.0006
Synovial infiltrate	-9.67	5.45	-1.77	0.080
Cartilage Outerbridge	-1.07	0.73	-1.47	0.147
Duration of symptoms	-0.21	1.25	-0.17	0.869
Age	0.24	0.27	0.87	0.387
BMI	-1.62	0.67	-2.42	0.018
KOOS quality of Life				
(Intercept)	50.86	16.72	3.04	0.003
Synovial infiltrate	-1.57	4.23	-0.37	0.712
Cartilage Outerbridge	-1.01	0.57	-1.77	0.081
Duration of symptoms	0.67	0.97	0.69	0.493
Age	0.33	0.21	1.57	0.121
BMI	-0.72	0.52	-1.38	0.171
Pre-op VAS				
(Intercept)	3.06	2.22	1.38	0.172
Synovial infiltrate	1.61	0.56	2.87	0.005
Cartilage Outerbridge	0.02	0.07	0.21	0.833
Duration of symptoms	0.06	0.13	0.50	0.620
Age	0.01	0.03	0.52	0.607
BMI	0.005	0.07	0.07	0.945

Table S3. Multivariate analysis between ΔKOOS and ΔVAS and synovial infiltrate and other OA risk factors

ΔKOOS	Estimate	Std. Error	T Value	p-value
ΔKOOS total score				
(Intercept)	13.13	20.82	0.63	0.531
Synovial infiltrate	10.97	5.28	2.08	0.042
Cartilage Outerbridge	-0.55	0.71	-0.77	0.443
Duration of symptoms	-1.072	1.17	-0.91	0.365
Age	-0.24	0.26	-0.94	0.352
BMI	0.77	0.64	1.21	0.233
ΔKOOS symptoms				
(Intercept)	11.99	21.09	0.57	0.572
Synovial infiltrate	10.34	5.35	1.93	0.058
Cartilage Outerbridge	-0.35	0.72	-0.49	0.629
Duration of symptoms	-0.006	0.26	-0.02	0.982
Age	0.006	0.25	0.02	0.982
BMI	0.34	0.65	0.52	0.602
ΔKOOS pain				
(Intercept)	-7.05	21.27	-0.33	0.742
Synovial infiltrate	13.61	5.40	2.52	0.014
Cartilage Outerbridge	-1.17	0.72	-1.62	0.459
Duration of symptoms	-0.89	1.20	-0.75	0.459
Age	-0.06	0.26	-0.24	0.811
BMI	1.22	0.65	1.87	0.067
ΔKOOS Function, daily living				
(Intercept)	6.07	25.40	0.24	0.812
Synovial infiltrate	9.86	6.45	1.53	0.132
Cartilage Outerbridge	-0.22	0.87	-0.25	0.804
Duration of symptoms	-0.96	1.43	-0.67	0.505
Age	-0.12	0.31	-0.37	0.710
BMI	0.56	0.78	0.72	0.475
ΔKOOS Sport				
(Intercept)	39.07	27.88	1.40	0.167
Synovial infiltrate	15.15	7.08	2.14	0.037
Cartilage Outerbridge	-0.66	0.95	-0.69	0.491
Duration of symptoms	-0.09	1.57	-0.06	0.954
Age	-0.92	0.34	-2.67	0.010
BMI	1.52	0.86	1.78	0.081
ΔKOOS quality of Life				
(Intercept)	57.15	27.17	2.10	0.040
Synovial infiltrate	6.28	6.90	0.91	0.366
Cartilage Outerbridge	-0.55	0.92	-0.59	0.556
Duration of symptoms	-1.46	1.53	-0.96	0.343
Age	-0.77	0.34	-2.29	0.026
BMI	0.54	0.83	0.65	0.519
ΔVAS				
(Intercept)	0.17	3.11	0.05	0.957

Synovial infiltrate	-1.91	0.79	-2.42	0.019
Cartilage Outerbridge	0.09	0.11	0.86	0.390
Duration of symptoms	0.14	0.17	0.80	0.424
Age	-0.002	0.04	-0.06	0.955
BMI	-0.07	0.09	-0.72	0.473

KOOS = Knee Injury and Osteoarthritis Outcome Score; VAS = visual analogue score, BMI = body mass index, Δ = post-operative – pre-operative.

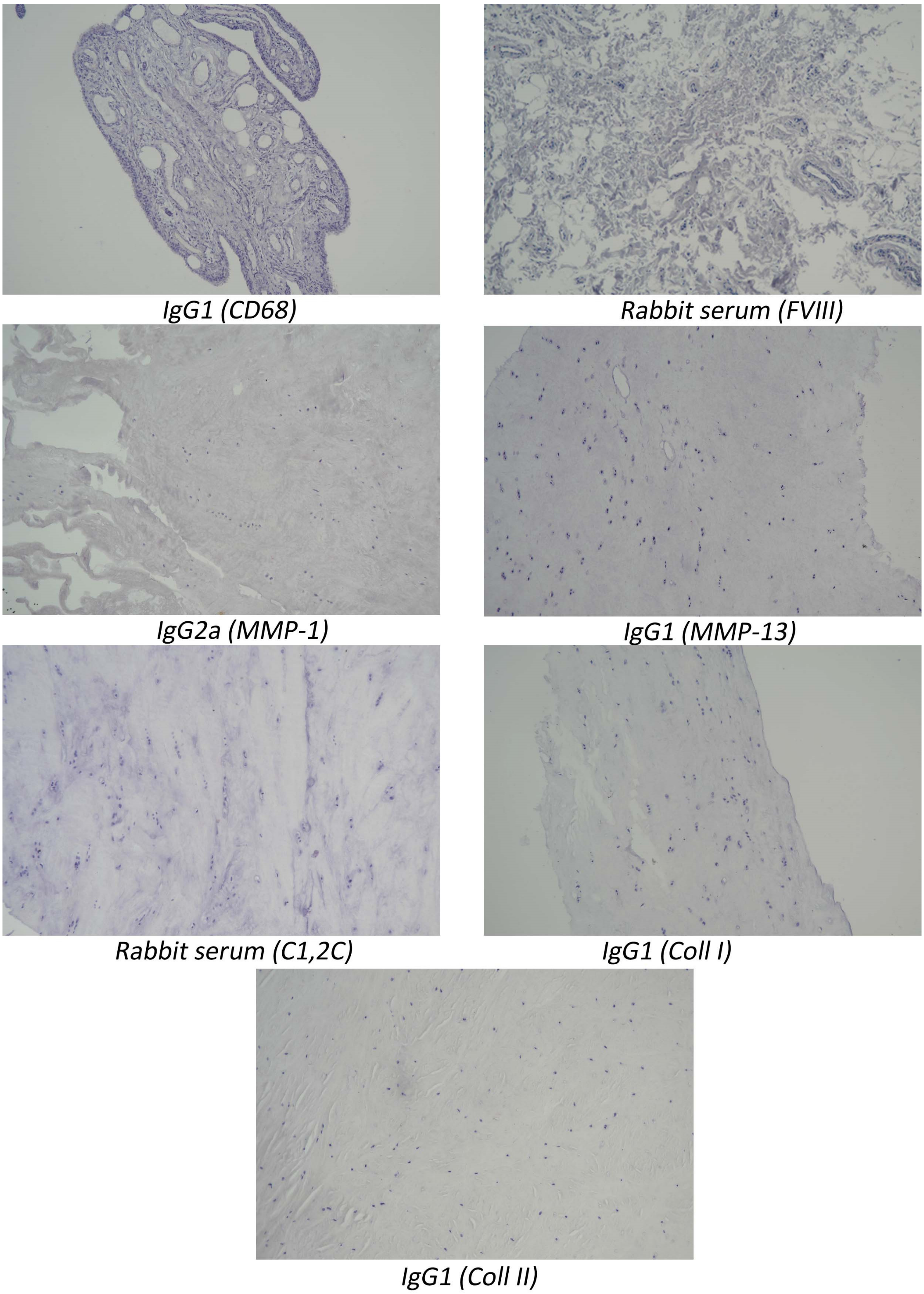

Figure S2. Immunohistochemistry of Isotype control. Each different image showed the isotype staining at the same concentration used for each marker analyzed as indicated by labels.
